# Late-onset Cytomegalovirus Infection Associated With Gastric Outlet Obstruction in a Preterm Twin

**DOI:** 10.1097/PG9.0000000000000025

**Published:** 2020-11-11

**Authors:** Verena Dries, Dirk Müller, Franz G. Schnekenburger, Irina Berger, Robert Rottscholl, Andreas C.W. Jenke

**Affiliations:** From the * Department of Neonatology and Paediatric Gastroenterology, Children´s Hospital Kassel, Klinikum Kassel, Germany; † Department of Paediatric Surgery, Children´s Hospital Kassel, Klinikum Kassel, Germany; ‡ Department of Pathology, Klinikum Kassel, Germany; § University of Witten/Herdecke, Germany.

**Keywords:** cytomegalovirus, gastric outlet obstruction, very low birth weight infant

## Abstract

The infant was born at a gestational age of 28 + 2 weeks as second twin to a 26-year-old woman, G1/P0, due to eclampsia. The patient developed well and was on full oral feeds when he started to develop nonbilious vomiting at 5 weeks. He was diagnosed with pyloric hypertrophy and underwent pylorotomy, but the condition did not improve and the patient was referred to our hospital. Here, esophagogastroduodenoscopy showed severely inflamed esophageal and gastric mucosa which was found to be due to cytomegaly virus (CMV) infection and a nonpassable pylorus. The patient underwent pyloroplasty revealing a fibrous pyloric ring. Histology showed giant cells suggestive of CMV infection which was confirmed by polymerase chain reaction. He was started on valganciclovir and discharged 4 weeks later on full enteral feeds. To our knowledge, this is the first case of gastric outlet obstruction due to CMV infection in a premature infant.

Cytomegalovirus (CMV), a double-stranded DNA virus belonging to the family of Herpesviridae, is the most common cause of congenital and postnatal virus infection. After primary infection which often presents either asymptomatic or as flu-like illness, the virus can persist in hematopoietic stem cells and monocytes causing latent infection. Particularly, young children who acquire the infection <3 years of age are shedding high amounts of virus through saliva and urine lasting in some cases even until the age of 7 years. Also, seropositive women can secrete reactivated CMV into breastmilk. Thus, pregnant women and young children are particularly exposed to CMV. Whereas congenital CMV infection not infrequently causes severe neurological disease (^1^), postnatal infection is generally mild or even asymptomatic. Nevertheless, it still holds a great risk to newborns with a gestational age <32 weeks and those with very low birth weight (<1500 g). Typical findings in these vulnerable groups of patients are pneumonia, thrombocytopenia, hepatitis, colitis, and hearing impairment (^2^), but to date, no case of gastric outlet obstruction has been described in this group of patients.

## CASE REPORT

The patient was born in a peripheral hospital as second twin with a gestational age of 28 + 2 weeks and a birthweight of 1325 g to a 26-year-old woman, G1/P0, due to preeclampsia. Cardiorespiratory adaption was uncomplicated and the patient was stabilized with CPAP at low levels of oxygen. During the following weeks, respiratory support was gradually weaned and enteral feeding was initiated with breastmilk which was pasteurized until the patients gestational age was 32 weeks. At the age of 6 weeks, the patient was on full enteral feeds without any respiratory support when he developed nonbilious vomiting. Eventually, he did not tolerate any oral feeding and received total parental nutrition. Ultrasound was reported to have revealed a hypertrophy and stenosis of the proximal part of the pylorus, which had not the classic appearance of pyloric hypertrophy. Nevertheless, the patient underwent pyloromyotomy, but the condition did not improve. He still did not tolerate any oral feeding and remained dependent on total parenteral nutrition. Thus, at the age of 9 weeks, the patient was transferred to our hospital for further diagnostic work-up and therapy. At the same time, his older twin was discharged home.

**FIGURE 1.**
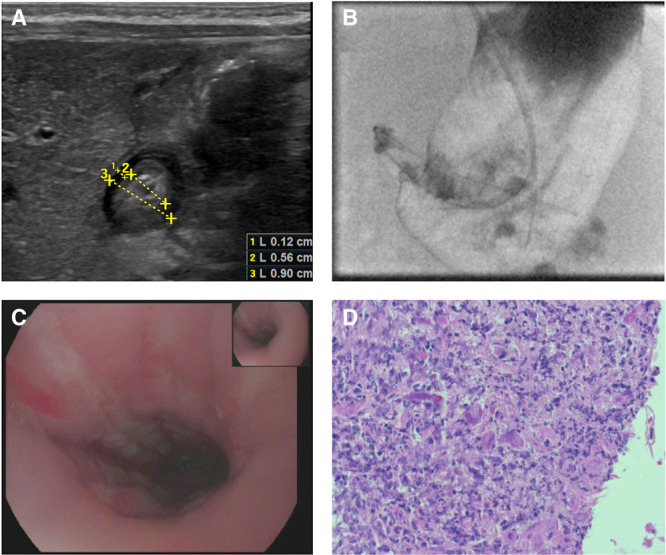
Diagnostics and histology. A, Ultrasound of the pylorus before second surgical intervention demonstrating hypertrophy and stenosis of the proximal part of the pylorus. B, X-ray swallow examination showing a nonpassable pylorus and dilated stomach. C, EGD showing severely inflamed mucosa of the esophagus. D, Histology material deriving from the stomach showing giant cells (arrow), typically associated with virus infections. EGD = esophagogastroduodenoscopy.

Initially, we performed an ultrasound demonstrating and stenotic pylorus (Fig. 1A), which was confirmed by a functional x-ray swallow examination showing a nonpassable pylorus and dilatated stomach (Fig. 1B). The esophagogastroduodenoscopy revealed a severely inflamed gastric and esophageal mucosa (Fig. 1C) and again a non-passable pylorus. Initially, we related this chemical stress due to the gastric obstruction, but histology showed giant cells (Fig. 1D) typical for CMV infection which was confirmed by polymerase chain reaction. The patient was started on intravenous ganciclovir which was later switched to oral valganciclovir and omeprazole. To analyze the extent of the CMV infection blood, urine and spinal fluid was tested. Whereas high viral loads were found in urine and blood, no virus could be detected in the spinal fluid (Table 1). Also, brain ultrasound, ophthalmological assessment, and brainstem evoked response audiometry did not show any signs of cerebral involvement. Also electrolytes, transaminases, complete blood count, coagulation as well as albumin were all in within normal limits. Thus, apart from the gastrointestinal tract, no other organ system seemed to be involved. Moreover, to determine whether this infection was congenital or postnatally acquired, we analyzed the dry blood sample obtained for screening for inborn errors of metabolism on the third day of life for CMV, which was found negative ruling out a congenital infection.

**TABLE 1.** CMV quantitative PCR

	Patient	Normal values
Urine (IU/ml)	>900,000	<200
EDTA-blood (IU/ml)	1249	<200
Gastric/esophageal tissue	Positive	Negative
Spinal fluid (IU/ml)	<100	<200

CMV = cytomegalovirus; EDTA = ethylenediaminetetraacetic acid; PCR = polymerase chain reaction.

Due to the almost complete gastric outlet obstruction, we performed an explorative laparotomy. Intraoperatively, the gastric mucosa just before the pylorus appeared normal with a submucous fibrotic ring extending into the proximal one-third of the pylorus. After resection of this ring, a pyloroplasty according to Heineke-Mikulicz was performed. Histopathology of the resected tissue showed giant cells typical CMV infection. Using nested multiplex-polymerase chain reaction CMV-DNA (^3^) could be amplified in the resected tissue as well as esophageal and gastric biopsies (Table 1). On day 2 after surgery, continuous enteral feeding via a duodenal tube was initiated and gradually increased until full enteral feeding was achieved on day 12 after surgery. We then gradually switch to oral feeding via gastric tube and then by mouth as tolerated reducing the duodenal tube feeding in parallel. Initial swallowing difficulties were addressed by logopaedic therapy. Three weeks after surgery, we performed a control esophagogastroduodenoscopy which showed good passage of the pylorus but still moderate to severe inflammation of the esophagus. Thus, we decided to extend the therapy with valganciclovir for 3 months and omeprazole for 6 weeks. The gastric and duodenal tube was removed and the patient was discharged home on day 26 after surgery at a gestational age of 41 weeks with a weight of 3410 g (26th percentile).

## DISCUSSION

Here, we describe the first case of noncongenital neonatal gastric outlet obstruction in a newborn infant associated with intestinal CMV infection. In our case, oral feeding was tolerated well until 6 weeks of age making a congenital malformation such as a duodenal web very unlikely. Moreover, the breastmilk was initially pasteurized because the mother was seropositive for CMV-IgG positive while the infants gestational age was <32 weeks (^2^). Pasteurization was only stopped 2 weeks prior the development of the nonbilious vomiting. Because we ruled out congenital infection, it is very likely that the infection occurred at that time. Also, ultrasound and pathology of the resected pylorus showed no muscular hypertrophy, but a fibrous tissue with active CMV replication further strengthening the idea of a causal relationship between intestinal CMV infection and gastric outlet obstruction in this patient. Because no common signs of CMV infection (e.g. elevated liver enzymes) could be detected, we believe that CMV infection was mainly restricted to the intestine. Interestingly, in the literature, we could not find any reports on neonatal gastrointestinal CMV infections associated with isolated gastritis, esophagitis or even gastric outlet obstruction. Only reports on CMV identified in tissue samples from colonic strictures following NEC surgery have been published so far (^4^). However, in adult immunocompromised CMV-naïve patients, CMV gastritis and esophagitis is a well-described complication (^5,6^), and also some cases with gastric outlet obstruction due to local intestinal inflammatory responses to CMV have been reported (^4,7,8^). Still it remains speculative, if the intestinal stricture was the result of a primary viral infection or a secondary infection in a transitory immunodeficiency due to enterocolitis (^9,10^).

Overall, this case adds to the variety of symptoms in CMV infections and underscores the necessity to always keep CMV as a possible cause of intestinal symptoms in mind as neonatologist or gastroenterologist.

The study was performed in accordance with the Declaration of Helsinki. Consent from legal parents was obtained to publish the individual data.
